# Finding Correlations Between mRNA and Protein Levels in *Leishmania* Development: Is There a Discrepancy?

**DOI:** 10.3389/fcimb.2022.852902

**Published:** 2022-07-12

**Authors:** Leonardo Cortazzo da Silva, Juliana Ide Aoki, Lucile Maria Floeter-Winter

**Affiliations:** Laboratório de Fisiologia de Tripanossomatídeos, Departamento de Fisiologia, Instituto de Biociências, Universidade de São Paulo, Sao Paulo, Brazil

**Keywords:** metacyclogenesis, amastigote differentiation, life cycle, gene expression, transcriptome, proteome

## Abstract

Multiple genes and proteins have been identified as differentially expressed in the stages of the *Leishmania* life cycle. The differentiation processes are implicated in specific transcriptional and proteomic adjustments driven by gene expression regulation mechanisms. *Leishmania* parasites lack gene-specific transcriptional control, and gene expression regulation mostly depends on posttranscriptional mechanisms. Due to the lack of transcriptional regulation, criticism regarding the relevance of transcript quantification as a possible and efficient prediction of protein levels is recurrent in studies that use transcriptomic information. The advent of high-throughput technologies has improved the analysis of genomes, transcriptomes and proteomes for different organisms under several conditions. Nevertheless, defining the correlation between transcriptional and proteomic profiles requires arduous and expensive work and remains a challenge in *Leishmania*. In this review, we analyze transcriptomic and proteomic data for several *Leishmania* species in two different stages of the parasite life cycle: metacyclogenesis and amastigogenesis (amastigote differentiation). We found a correlation between mRNA and protein levels of 60.9% and 69.8% for metacyclogenesis and amastigogenesis, respectively; showing that majority mRNA and protein levels increase or decrease concomitantly. Among the analyzed genes that did not present correlation indicate that transcriptomic data should be carefully interpreted as protein expression. We also discuss possible explanations and mechanisms involved for this lack of correlation.

## Introduction

The central dogma of biology, as described by Francis Crick (Crick, 1970), provided a solid comprehension of the genetic flow followed by most cells, despite its limitations. The idea that genetic information flows from DNA to RNA molecules *via* transcription and from RNA to proteins *via* translation is still being vastly explored in molecular biology practices (Li and Xie, 2011; Liu et al., 2018; Schneider-Poetsch and Yoshida, 2018). The latest advances in genomics, transcriptomics and proteomics have enabled assessing levels of gene and protein expression in cells under different conditions (Kim et al., 2010; Zhang et al., 2014; Segundo-Val and Sanz-Lozano, 2016; Aslam et al., 2017; Hung and Weng, 2017). Considering that several mechanisms are involved in transcription and translation regulation, inferences can be made to extrapolate one type of data to predict the other, even though there is no trivial relationship between levels of transcripts and proteins (Jansen et al., 2002; Greenbaum et al., 2003; De Sousa Abreu et al., 2009; Buccitelli and Selbach, 2020).

In most eukaryotes, known regulatory mechanisms present monocistronic mRNAs with transcription regulated by individual promoters, enhancers and transcription factors (Cramer, 2019) resulting in highly regulated RNA production. Some of these mechanisms, however, are not present in trypanosomatids such as *Leishmania*, which exclusively utilizes posttranscriptional regulation (Martínez-Calvillo et al., 2004; Ivens et al., 2005; Clayton, 2016; De Pablos et al., 2016; Clayton, 2019). The absence of gene-specific transcription regulation raises questions about the relevance of quantifying mRNA in these organisms: How could the quantification of mRNA provide insight on the orchestration of the phenotype in different scenarios? What could be the relevance of measuring mRNA levels in these organisms? How is mRNA information relevant for overall expression analysis?

*Leishmania* is a protozoan parasite and causative agent of leishmaniases, a group of diseases characterized by cutaneous, mucocutaneous or visceral lesions. Leishmaniases is caused by at least 20 species of the *Leishmania* genus, affecting approximately 0.7 to 1 million people every year in nearly 100 endemic countries (Burza et al., 2018). According to the World Health Organization (WHO), leishmaniases are emerging tropical neglected diseases for which new treatments should be prioritized (Burza et al., 2018).

**Figure 1:**
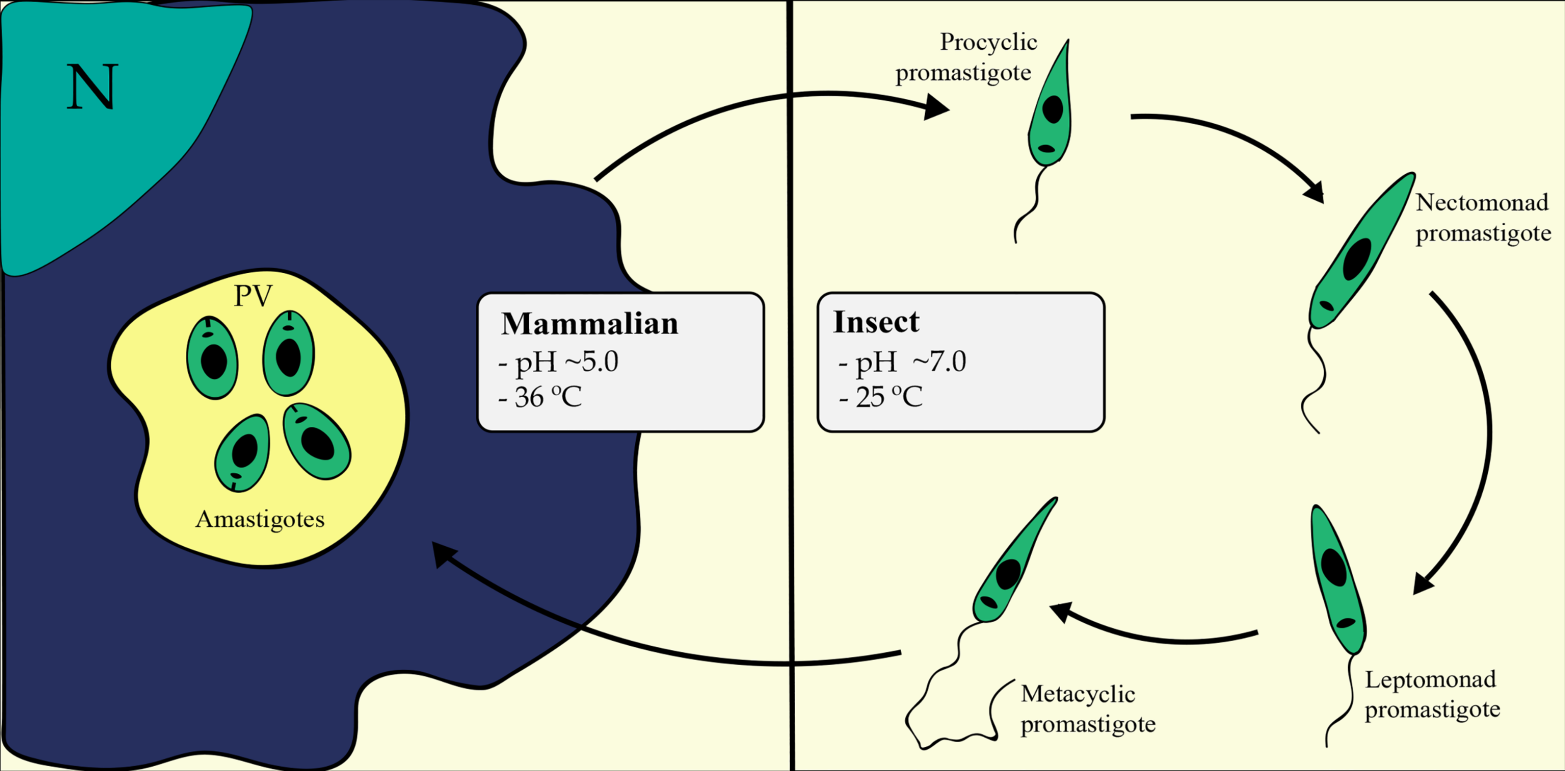
*Leishmania* life cycle in mammalian and insect hosts. The differentiation processes involve drastic changes in pH, temperature, and nutrient availability, challenging the parasite’s ability to orchestrate its gene expression accordingly. Metacyclogenesis is essentially represented in the right portion of the figure, comprising the differentiation from procyclic into nectomonad and then into leptomonad and metacyclic forms inside the sandfly digestive tract. Amastigogenesis is represented in the left portion of the figure, comprising the differentiation from metacyclic promastigote to amastigote forms inside the mammalian host macrophage. N, Macrophage Nucleus; PV, Parasitophorous Vacuole.

The parasite presents two different life forms: the promastigote and the amastigote (**Figure 1**). Drastic changes in pH, temperature and nutrient availability are related to differentiation (Gupta et al., 2001); these changes challenge the parasite’s ability to adapt under a lack of regulation at the transcriptional level. The promastigote forms present a motile elongated body with an apparent flagellum, live and multiply within the digestive tract of sandflies (25°C and pH ~7.0) in a microenvironment containing insect gut nutrients, digestive enzymes associated with its microbiota and saliva components (Kamhawi, 2006; Dostálová and Volf, 2012). In this environment, the promastigote undergoes a series of morphological changes that culminate in differentiation into metacyclic forms, which are the infectious and nonreplicative stage. This process includes the differentiation of procyclic promastigotes into nectomonad promastigotes, leptomonad promastigotes and metacyclic promastigotes (Sacks and Perkins, 1984; Bates and Tetley, 1993; Serafim et al., 2012). Metacyclic promastigotes are defined as highly infective, rapidly swimming, nonproliferative and present a long flagellum that allows motility to infect a mammalian host (Sacks and Perkins, 1984). Metacyclic promastigotes infect the host during the sandfly blood meal and differentiate into amastigotes once inside host cells. Additionally, new insight into parasite development have been described and have led to a revised *Leishmania* life cycle (Bates, 2018). Furthermore, metacyclic promastigote forms are able to dedifferentiate in the sandfly, enhancing parasite population growth through a second blood meal and providing a greater disease transmission potential (Serafim et al., 2018).

Once inside mammalian host cells, metacyclic promastigotes differentiate into amastigotes. The amastigote stage of *Leishmania* displays a rounded cell body with a nonapparent flagellum (Howells and Gardener, 1972; Gardener et al., 1973; Hentzer and Kobayasi, 1977). These life forms live and multiply inside the host phagocytic compartment (36°C and pH ~5.0).

The advent of high-throughput technologies has improved the analysis of genomes, transcriptomes and proteomes; however, establishing a correlation among these profiles is still a challenge. Most studies focus individually on mRNA or protein expression, as performing both can be time-consuming, expensive and demands trained personnel. Despite that, it is worth noting that general trends in groups of genes observed in transcriptomes or proteomes are not predictive of individual gene or protein expression. Therefore, the present review aims to discuss the correlation between mRNA and protein levels in the *Leishmania* life cycle, mainly with regard to metacyclogenesis and amastigote differentiation.

### mRNA *Versus* Protein Studies in *Leishmania*

**Figure 2:**
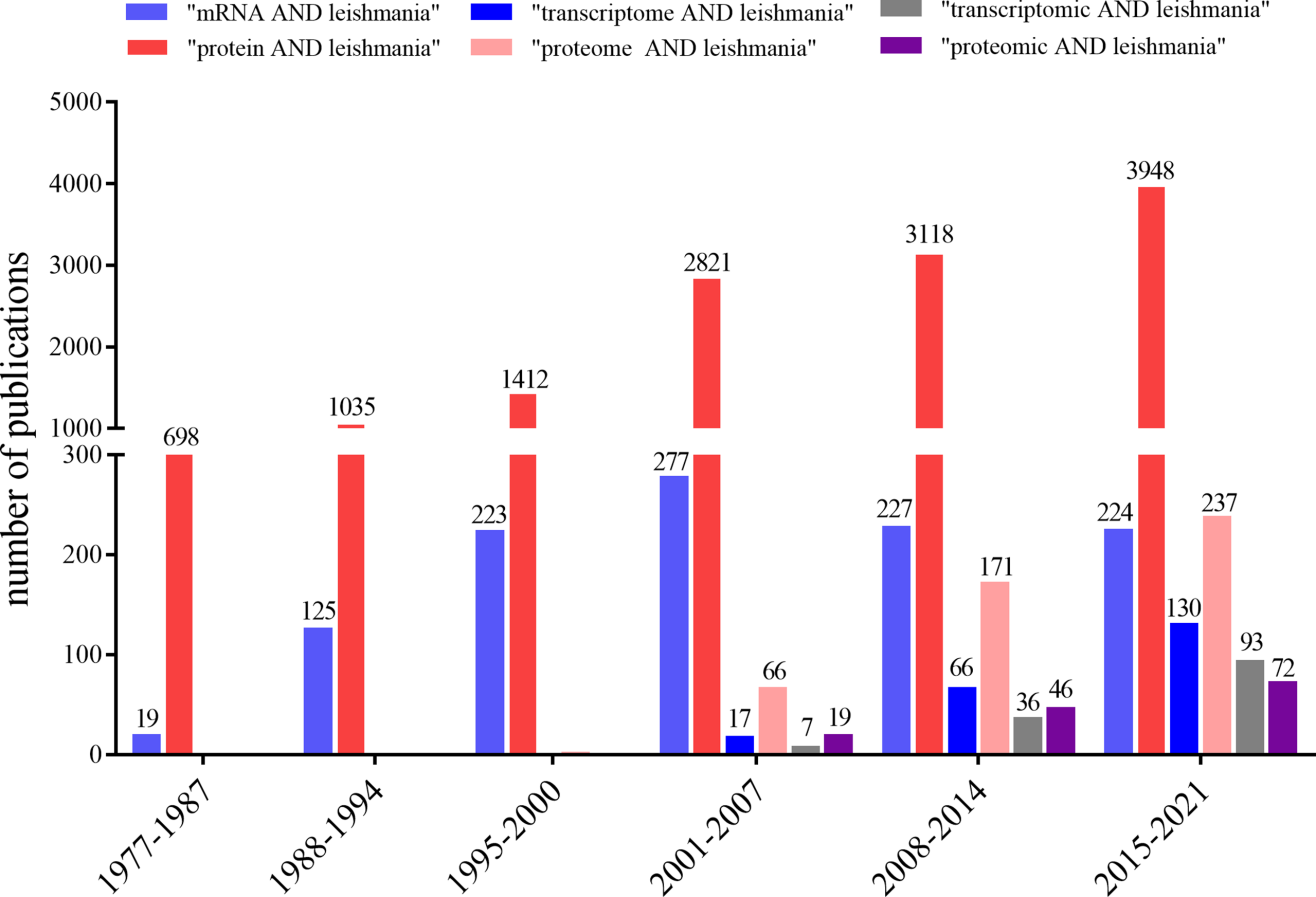
Graph showing the number of publications retrieved in PubMed using the terms “mRNA AND leishmania” (light blue bars), “protein AND leishmania” (red bars), “transcriptome AND leishmania” (dark blue bars), “proteome AND leishmania” (pink bars), “transcriptomic AND leishmania” (gray bars), and “proteomic AND leishmania” (purple bars). The searches were conducted up to December 2021.

To understand the extent of *Leishmania* mRNA and protein studies, we conducted a simple search in PubMed using the keywords “mRNA”, “transcriptome”, “transcriptomic”, “protein”, “proteome”, “proteomic”, “leishmania” and the Boolean operator “AND”. Considering that transcriptomic is defined as a methodology to study transcriptome which is a set of mRNAs; and it is complementary to proteomic that is defined as a methodology to study proteome which is a set of proteins, we encompassed the number of publications up to December 2021 using these terminologies (**Figure 2**).

A significant difference between the Boolean pairs was identified: 1,095 publications for “mRNA AND leishmania” *versus* 13,032 publications for “protein AND leishmania”, indicating an order of magnitude in favor of protein researches. The advent of new sequencing technologies starting in early 2000´s has generated studies on a large scale and the discrepancy decreased significantly, showing 213 publications for “transcriptome AND leishmania” *versus* 474 publications for “proteome AND leishmania”, and 136 publications for “transcriptomic AND leishmania” *versus* 137 publications for “proteomic AND leishmania” (**Figure 2**). Based on these findings, the question that we are approaching in this review remains open: how relevant is to quantify mRNA or protein, individually, to provide a phenotype pattern?

### Transcription and mRNA Processing in *Leishmania*

**Figure 3:**
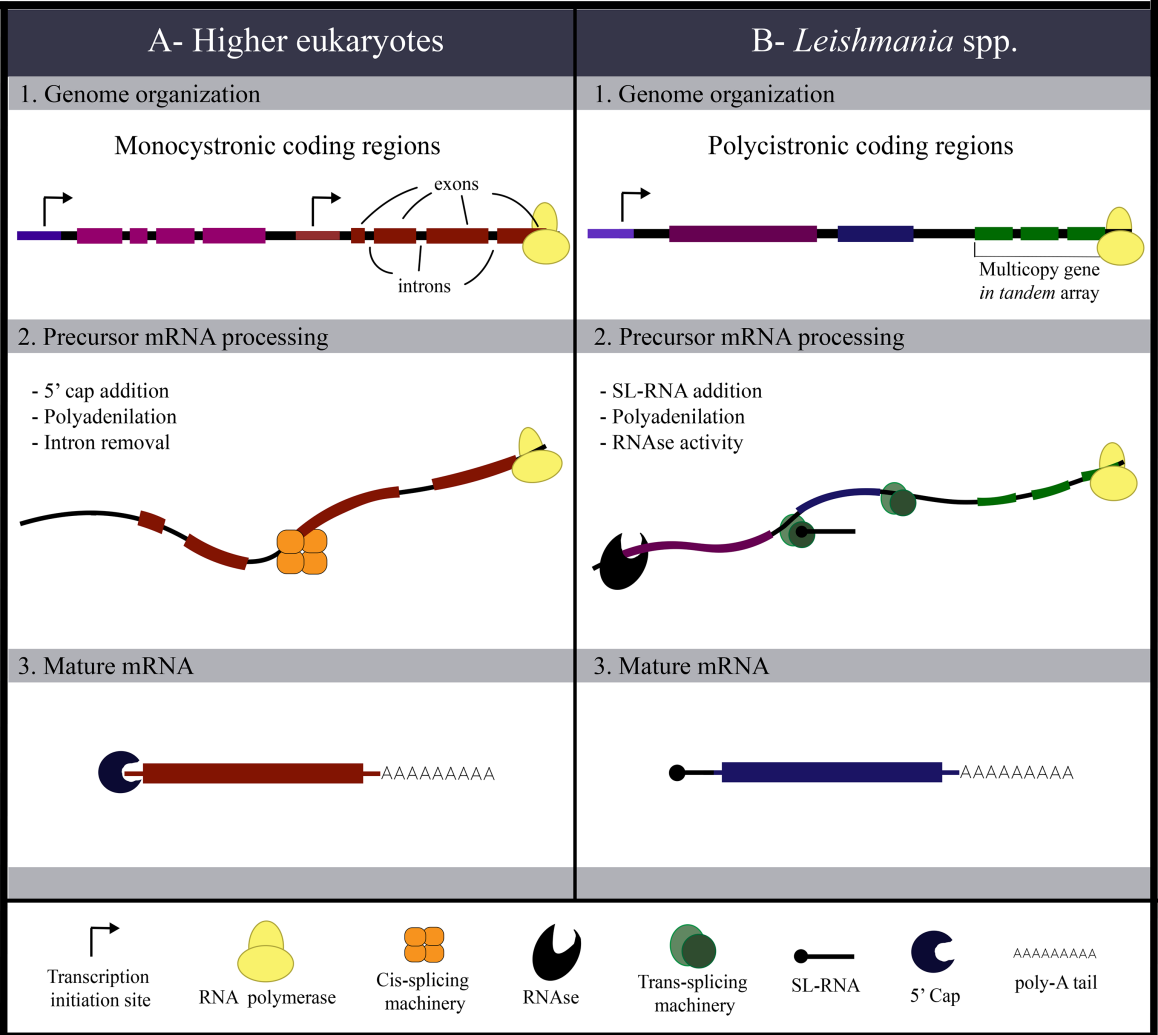
Main aspects of polycistronic and monocistronic gene expression. **(A)** Monocistronic coding regions are common to most higher eukaryotes. With this genome organization, protein-coding genes are organized within intronic portions that are nonprotein-coding regions. Transcription is regulated by promoters and enhancers; thus, transcription is individually controlled. Processing of precursor mRNA involves the removal of introns, 5’ cap addition and polyadenylation, generating the mature mRNA molecule that is translated after export from the nucleus. **(B)** The polycistronic organization of coding genes differentiates *Leishmania* gene expression. The absence of transcriptional control is shown, with several genes encoded in the same polycistronic coding region being transcribed together. Another difference is the existence of multicopy genes in tandem arrays. All these characteristics point to uncontrolled transcription. Processing of precursor mRNA involves *trans*-splicing (addition of the SL molecule), polyadenylation and RNAse activity to eliminate mRNAs that will not be translated.

Protein-coding genes in the *Leishmania* genome are organized into long polycistronic units containing multiple open reading frames, as observed in chromosome 1 (containing 29 and 50 genes in each of the two polycistronic units) and chromosome 3 (containing 69 and 30 genes in each of the two polycistronic units) (Myler et al., 1999; Martínez-Calvillo et al., 2004). These units can be up to 100,000 bases long and have no introns. (Ivens et al., 2005; Clayton, 2016). The transcription of these units generates long precursor mRNAs that are processed cotranscriptionally, generating mature mRNAs corresponding to each gene originally encoded on the polycistronic unit (Liang et al., 2003; Martínez-Calvillo et al., 2004). Then, mRNAs are *trans-*spliced with the addition of a spliced leader (SL) molecule to the 5’ end of each mRNA (Clayton, 2016). The polyadenylation process occurs simultaneously, as the long polycistronic precursor is divided into smaller mature mRNAs encoding single genes to be translated (Michaeli et al., 1993; Liang et al., 2003; Michaeli, 2011; Preußer et al., 2012; Clayton, 2016). SL addition and polyadenylation enhance molecular stability and avoid degradation (Agabian, 1990), which can occur in the nucleus by RNase activity (Clayton, 2019). In this context, competition between mRNA degradation and processing is relevant to the levels of mature mRNAs in the cell (Fadda et al., 2014). Next, individual mature mRNAs are exported from the nucleus (Bühlmann et al., 2015). In the cytosol, the binding of eukaryotic translational initiation factors recruits ribosomes and initiates translation (Clayton, 2019) (**Figure 3**). Knockout of different translation initiation factors in *Leishmania* impacts the cells’ whole proteome, affecting parasite morphology and infectivity (Tupperwar et al., 2019; Baron et al., 2020; Shrivastava et al., 2021). Posttranscriptional and posttranslational controls, including mRNA stability, translation initiation and protein folding are mechanisms of gene expression regulation in *Leishmania*, indicating how complex the process from gene to protein can be (Clayton and Shapira, 2007; Haile and Papadopoulou, 2007; Stiles et al., 2016).

In contrast, gene expression in higher eukaryotes is monocistronic, with coding regions individualized by the presence of gene transcription promoters and enhancers. Transcription factors activate different transcriptional programs, modulating the cellular response to environmental cues. Premature RNA processing in higher eukaryotes involves the removal of intronic regions after the addition of the 5’ cap, followed by poly-A tail synthesis (**Figure 3**), and these processes are generally coupled (Maniatis and Reed, 2002).

### Involvement of Noncoding RNAs and RNA Binding Proteins in the *Leishmania* Life Cycle

Recently, new mechanisms of regulation have been described to play important roles in *Leishmania* gene expression (Nandan et al., 2017; Azizi and Papadopoulou, 2020). This fact highlights that *Leishmania* possesses tightly tuned regulation of its gene expression, though the regulatory elements themselves are still largely uncharacterized (Fernandes et al., 2019). Among these, noncoding RNAs (ncRNAs) and RNA-binding proteins (RBPs) may contribute to the mRNA-protein level discrepancies observed in *Leishmania*.

ncRNAs vary in size and have many different mechanisms of action. In association with other molecules, ncRNAs control transcription, translation and RNA degradation (Mattick, 2001; Hombach and Kretz, 2016; Fernandes et al., 2019). Some *L. major* and *L. donovani* untranslated regions (UTRs) have been identified as origins of ncRNAs (Castro et al., 2017). Thousands of ncRNAs of different classes were also identified in the *L. braziliensis* genome and confirmed by RNA-seq analysis, suggesting that they are real transcripts (Torres et al., 2017; Ruy et al., 2019). Additionally, RNA-seq data have revealed a ncRNA among the top five differentially expressed transcripts in comparison of two *L. amazonensis* lineages, indicating an important role in the modulation of a specific metabolic pathway, such as arginase activity (Aoki et al., 2017). Developmentally regulated ncRNAs specific to the amastigote life stage of *L. donovani* present similar transcription characteristics to known protein-coding mRNAs (Dumas et al., 2006).

RBPs also play an important role in regulating translation in trypanosomatids and have been described as important regulatory components in *Leishmania* (Terrão et al., 2017; Ruy et al., 2019). RBPs interact with mRNAs, representing another posttranscriptional mechanism that regulates gene expression. The association of RBPs with methyltransferases suggests the role of these proteins in regulating arginine methylation, a known posttranslational modification of proteins (Ferreira et al., 2014). The relationship between RBPs and methyltransferases also impact on the virulence and protein stability of *Leishmania* (Ferreira et al., 2020). There is an evidence of a potential protective effect of RBPs in *L. mexicana*, accumulating in the nucleus in response to actinomycin D treatment (Názer and Sánchez, 2011).

The regulatory elements described in this session have not been fully explored and described in *Leishmania* yet, probably due to the complexity of their mechanisms and interactions, and also the lack of sufficient genome annotations. However, characterization of these elements might improve knowledge of the biology of the parasite, genomic organization, and regulation processes, providing potential new biomarkers and/or drug targets.

The *Leishmania* genome appears to be constitutively expressed at the transcriptional level (Leifso et al., 2007; Alcolea et al., 2019), presenting a small percentage of differentially expressed mRNAs between life stages (Holzer et al., 2006; Cohen-Freue et al., 2007; Saxena et al., 2007; Aoki et al., 2017). A previous comparative analysis of proteomes and transcriptomes of *L. infantum* during amastigogenesis revealed that although expression trends were comparable, fold changes usually did not correlate (McNicoll et al., 2006). To better understand the extent to which mRNA and protein data are connected in *Leishmania*, we reviewed published studies describing mRNA and protein expression during *Leishmania* development to find correlations focusing on selected genes and proteins related to differentiation of parasite forms during the life cycle.

## Metacyclogenesis

Metacyclogenesis is essentially the process of differentiation from procyclic to metacyclic promastigotes. Throughout this process, procyclic promastigotes differentiate into nectomonad and leptomonad promastigote stages, finally becoming metacyclic promastigotes (Sacks and Perkins, 1984; Bates and Tetley, 1993; Sacks and Kamhawi, 2001; Serafim et al., 2012). At the proteomic level, metacyclic promastigotes present a reduction in the abundance of proteins involved in protein synthesis and an increase in proteins involved in cell motility (Mojtahedi et al., 2008; Amiri-Dashatan et al., 2020b). Additionally, transcriptomic analysis of metacyclogenesis has demonstrated that each promastigote life stage has exclusive differentially expressed transcripts associated with them (Inbar et al., 2017; Coutinho-Abreu et al., 2020).

Overall, the specific conditions that trigger metacyclogenesis have not been fully disclosed. In nature, this process occurs within the midgut of the sandfly and involves stress induction and a lack of nutrients (Bates and Tetley, 1993; Kamhawi, 2006; Bates, 2008). Furthermore, the absence of purines seems to play a role in triggering metacyclogenesis (Serafim et al., 2012). Binding to the midgut of the insect is an essential process for promastigote development and this binding capability is strictly stage specific: it is observed in the leptomonad and nectomonad uninfective promastigote forms but it is not common in the metacyclic stage (Pimenta et al., 1992; Wilson et al., 2010). Surface glycoconjugate lipophosphoglycan (LPG) has been described as responsible for host midgut binding in *L. major* (Pimenta et al., 1992) and it is also hypothesized to be the molecule responsible for midgut binding in other *Leishmania* species (Sacks and Kamhawi, 2001). Detachment and release from the host midgut occurs as the LPG molecule becomes elongated and modified by transferase enzymes (McConville et al., 1992; Novozhilova and Bovin, 2010). The observed stage-specific ability to bind to the host midgut found in different *Leishmania* species (Pimenta et al., 1992; Wilson et al., 2010) is a finding that might indicate that the expression pattern of mRNAs and proteins involved in LPG synthesis is accordingly regulated.

Since the host midgut microenvironment plays an important role in metacyclogenesis, questions on the reliability of cultured promastigotes as a model, compared to sandfly-derived promastigotes, have emerged (Alcolea et al., 2016). The insect microenvironment contains several digestive enzymes and a microbiota. Some stage-specific molecules have been identified to play a role in protecting the parasite from proteolytic activity found within the insect midgut (Sacks and Kamhawi, 2001). Other molecules present in the saliva of the insect are also crucial to the development of infection in the mammal and are capable of suppressing the host immune response and determining the fate of the infection (Katz et al., 2000; Andrade et al., 2007). Transcriptomic comparisons among sandfly and culture-derived metacyclic promastigotes have revealed an overall transcriptional similarity, with specific differences in transcripts associated with nutrient stress (such as amino acid transport, fatty acid biosynthesis, catabolism of ketone bodies, and protein recycling *via* autophagy) that appear upregulated in cultured metacyclic promastigotes (Inbar et al., 2017).

To assess correlations between mRNA and protein levels in metacyclogenesis, we analyzed data from 6 different studies, 3 of which contained transcriptomic data (Almeida et al., 2004; Dillon et al., 2015; Inbar et al., 2017) and 3 of which contained proteomic data (Mojtahedi et al., 2008; Amiri-Dashatan et al., 2020a; Amiri-Dashatan et al., 2020b) from *L. major* and *L. tropica*, differentiating from procyclic to metacyclic promastigotes. To standardize our comparisons, we used the *L. major* Friedlin genome as a reference for gene IDs. Even though there are obvious differences in transcriptomic and proteomic profile between these two *Leishmania* species, the authors decided to use gene IDs of *L. major* since it is the most complete genomic database among *Leishmania* species. In cases in which the reference genome was another, we obtained syntenic orthologs of the *L. major* Friedlin genome using TryTrip database (www.tritrypdb.org). It is also worth noting that our search for studies upon which to base the present work retrieved a significantly smaller number of proteomic and transcriptomic studies of *Leishmania* metacyclogenesis in comparison to those focusing on amastigote differentiation. All analyzed data was statistically verified by each individual study then, the authors of this review did not interfere with or perform own statistical analysis of the data used. It is important to take into consideration that different studies may have presented different statistical thresholds to determine the own differentially expressed genes and proteins. Because of this limitation, the results observed here should be interpreted as proof-of-concept, since they were obtained from an extensive literature review and manual *ad-hoc* curation. The analysis was performed using a simple search tool in a table containing raw data from every analyzed study to find correlation between protein and mRNA trends. There is a consideration that some genes in the *Leishmania* genome can present the same coding region but differ in UTRs (Rastrojo et al., 2019). This aspect of the *Leishmania* genome organization is very complex and not discussed within the analyzed studies that were based solely on coding regions.

**Table 1** shows differentially expressed mRNAs and proteins according to transcriptomic and proteomic studies comparing procyclic and metacyclic promastigotes. For most genes, correlations between protein and mRNA levels were detected, whereas some genes show opposite trends in comparisons. We analyzed genes within the following biological groups: stress response, mitochondria, gene expression, energy metabolism and cell signaling. Hypothetical proteins and other biological functions were also considered (**Table 1**). The genes were grouped to facilitate the analysis. Gene Ontology was originally characterized in the respective study (Almeida et al., 2004; Mojtahedi et al., 2008; Dillon et al., 2015; Inbar et al., 2017; Amiri-Dashatan et al., 2020a; Amiri-Dashatan et al., 2020b).

**Table 1:** Common genes found in independent transcriptomic and proteomic data obtained from metacyclogenesis (procyclic vs. metacyclicpromastigotes) differentiation.

Biological group		Gene ID		Protein		mRNA levels (P→ M)			Protein levels (P→ M)			Correlation			Species (mRNA data)		Species (protein data)		References	
Stress response		LmjF.26.0800		Glutathione peroxidase		**↑**			**↑**			✔			*L. tropica*		*L. tropica, L. major*		Amiri-Dashatan et al., 2020a; Amiri-Dashatan et al., 2020b	
		LmjF.15.1040		Tryparedoxin peroxidase		**↑**			**↑**			✔			*L. tropica*		*L. tropica, L. major*		Amiri-Dashatan et al., 2020a; Amiri-Dashatan et al., 2020b	
		LmjF.33.2390		TRAP1/HSP75		**↓**			**↑**			**×**			*L. major*		*L. tropica*		Dillon et al., 2015; Inbar et al., 2017; Amiri-Dashatan et al., 2020a	
		LmjF.12.1130		Putative NADH:flavin oxidoreductase/NADH oxidase		**↓**			**↑**			**×**			*L. major*		*L. tropica*		Dillon et al., 2015; Amiri-Dashatan et al., 2020a	
Mitochondrial		LmjF.28.0490		Putative propionyl-coa carboxylase beta chain		**↑**			**↓**			**×**			*L. major*		*L. major*		Dillon et al., 2015; Inbar et al., 2017; Amiri-Dashatan et al., 2020b	
		LmjF.15.0280		Putative ribonucleoprotein p18, mitochondrial		**↓**			**↑**			**×**			*L. major*		*L. tropica*		Dillon et al., 2015; Amiri-Dashatan et al., 2020a	
		LmjF.26.1710		Putative cytochrome c oxidase subunit V		**↓**			**↓**			✔			*L. major*		*L. tropica*		Dillon et al., 2015; Amiri-Dashatan et al., 2020a	
		LmjF.25.1130		Putative cytochrome c oxidase VII		**↓**			**↓**			✔			*L. major*		*L. tropica*		Dillon et al., 2015; Amiri-Dashatan et al., 2020a	
		LmjF.35.4430		Putative mitochondrial phosphate transporter		**↓**			**↑**			**×**			*L. major*		*L. major*		Dillon et al., 2015; Amiri-Dashatan et al., 2020b	
		LmjF.35.1540		Putative reiske iron-sulfur protein		**↑**			**↑**			✔			*L. major*		*L. major*		Dillon et al., 2015; Amiri-Dashatan et al., 2020b	
Gene Expression		LmjF.32.0050		Protein transport protein sec13		**↓**			**↓**			✔			*L. major*		*L. major*		Mojtahedi et al., 2008; Dillon et al., 2015.	
		LmjF.18.0740		Putative elongation factor Tu		**↓**			**↓**			✔			*L. major*		*L. tropica*		Dillon et al., 2015; Amiri-Dashatan et al., 2020a	
		LmjF.35.3100		Putative ATP-dependent RNA helicase		**↓**			**↓**			✔			*L. major*		*L. tropica*		Inbar et al., 2017; Amiri-Dashatan et al., 2020a	
		LmjF.10.0870		Histone H3		**↓**			**↓**			✔			*L. major*		*L. tropica*		Inbar et al., 2017; Amiri-Dashatan et al., 2020a	
		LmjF.36.0180		Elongation factor 2		**↓**			**↓**			✔			*L. major*		*L. major*		Mojtahedi et al., 2008; Dillon et al., 2015; Amiri-Dashatan et al., 2020b	
		LmjF.34.0840		Elongation factor 1-beta		**↓**			**↓**			✔			*L. major*		*L. major*		Dillon et al., 2015; Amiri-Dashatan et al., 2020b	
		LmjF.21.1552		RNA helicase		**↓**			**↓**			✔			*L. major*		*L. major*		Mojtahedi et al., 2008; Dillon et al., 2015.	
		LmjF.19.1560		Inosine-5′-monophosphate dehydrogenase		**↓**			**↑**			**×**			*L. major*		*L. tropica*		Dillon et al., 2015; Amiri-Dashatan et al., 2020a	
		LmjF.17.0725		Guanosine monophosphate (GMP) reductase		**↓**			**↓**			✔			*L. major*		*L. tropica*		Dillon et al., 2015; Amiri-Dashatan et al., 2020a	
		LmjF.32.2950		Nucleoside diphosphate kinase		**↓**			**↑**			**×**			*L. major*		*L. major*		Dillon et al., 2015; Amiri-Dashatan et al., 2020b	
		LmjF.25.0490		Putative RNA-binding protein, UPB1		**↑**			**↓**			**×**			*L. major*		*L. major*		Dillon et al., 2015; Amiri-Dashatan et al., 2020b	
Energy metabolism		LmjF.16.0440		Putative fucose kinase		**↑**			**↓**			**×**			*L. major*		*L. tropica*		Dillon et al., 2015; Amiri-Dashatan et al., 2020a	
		LmjF.16.0440		Putative fucose kinase		**↑**			**↑**			✔			*L. major*		*L. major*		Dillon et al., 2015; Amiri-Dashatan et al., 2020b	
		LmjF.20.0100		Phosphoglycerate kinase C, glycosomal		**↑**			**↑**			✔			*L. tropica*		*L. tropica*		Amiri-Dashatan et al., 2020a	
		LmjF.20.0100		Phosphoglycerate kinase C, glycosomal		**↓**			**↑**			**×**			*L. major*		*L. major*		Dillon et al., 2015; Amiri-Dashatan et al., 2020b.	
		LmjF.35.0030		Pyruvate kinase		**↑**			**↑**			✔			*L. tropica*		*L. tropica, L. major*		Amiri-Dashatan et al., 2020a; Amiri-Dashatan et al., 2020b	
		LmjF.29.2510		ATP-dependent 6-phosphofructokinase		**↓**			**↓**			✔			*L. tropica*		*L. tropica*		Amiri-Dashatan et al., 2020a	
		LmjF.29.2510		ATP-dependent 6-phosphofructokinase			**↑**			**↓**			**×**		*L. major*		*L. tropica*		Dillon et al., 2015; Amiri-Dashatan et al., 2020a	
		LmjF.28.2910		Glutamate dehydrogenase			**↑**			**↑**			✔		*L. major*		*L. major*		Dillon et al., 2015; Inbar et al., 2017; Amiri-Dashatan et al., 2020b	
		LmjF.28.2910		Glutamate dehydrogenase			**↓**			**↓**			✔		*L. tropica*		*L. tropica*		Dillon et al., 2015; Inbar et al., 2017; Amiri-Dashatan et al., 2020b	
		LmjF.15.1010		Glutamate dehydrogenase			**↓**			**↑**			**×**		*L. major*		*L. tropica*		Inbar et al., 2017; Amiri-Dashatan et al., 2020a	
		LmjF.34.3670		Putative vacuolar ATP synthase catalytic subunit A			**↓**			**↓**			✔		*L. major*		*L. tropica*		Dillon et al., 2015; Amiri-Dashatan et al., 2020a	
		LmjF.19.0200		ATP/ADP translocase			**↓**			**↑**			**×**		*L. major*		*L. major*		Almeida et al., 2004; Dillon et al., 2015; Inbar et al., 2017; Amiri-Dashatan et al., 2020b	
		LmjF.18.0560		V-type proton ATPase subunit C			**↓**			**↓**			✔		*L. major*		*L. major*		Dillon et al., 2015; Amiri-Dashatan et al., 2020b	
Cell signaling		LmjF.25.1420		GTP-binding nuclear protein			**↓**			**↑**			**×**		*L. major*		*L. major*		Inbar et al., 2017; Amiri-Dashatan et al., 2020b	
		LmjF.29.2200		Putative GTP-binding protein			**↓**			**↓**			✔		*L. major*		*L. tropica*		Inbar et al., 2017; Amiri-Dashatan et al., 2020a	
Hypothetical proteins		LmjF.08.1100		Hypothetical protein			**↓**			**↓**			✔		*L. major*		*L. major*		Dillon et al., 2015; Amiri-Dashatan et al., 2020b	
		LmjF.32.0840		RNA binding protein DRBD18			**↓**			**↑**			**×**		*L. major*		*L. major*		Dillon et al., 2015; Amiri-Dashatan et al., 2020b	
Other		LmjF.29.0760		Putative lipophosphoglycan biosynthetic protein			**↓**			**↓**			✔		*L. major*		*L. tropica*		Inbar et al., 2017; Amiri-Dashatan et al., 2020a	
		LmjF.36.3910		S-Adenosylhomocysteine hydrolase			**↓**			**↓**			✔		*L. major*		*L. tropica, L. major*		Dillon et al., 2015; Amiri-Dashatan et al., 2020a; Amiri-Dashatan et al., 2020b	
		LmjF.17.0250		Cystathionine β-synthase			**↓**			**↓**			✔		*L. major*		*L. major*		Mojtahedi et al., 2008; Dillon et al., 2015.	
		LmjF.03.0200		Putative delta-1-pyrroline-5-carboxylate dehydrogenase			**↓**			**↓**			✔		*L. major*		*L. major*		Dillon et al., 2015; Amiri-Dashatan et al., 2020b	
		LmjF.11.0630		Putative aminopeptidase			**↓**			**↑**			**×**		*L. major*		*L. major*		Dillon et al., 2015; Amiri-Dashatan et al., 2020b	
		LmjF.11.0630		Putative aminopeptidase			**↓**			**↓**					*L. major*		*L. tropica*		Dillon et al., 2015; Amiri-Dashatan et al., 2020a	
		LmjF.35.2050		60S ribosomal protein L32			**↓**			**↑**			**×**		*L. major*		*L. major*		Dillon et al., 2015; Amiri-Dashatan et al., 2020b	
		LmjF.22.1410		Ca2+-binding EF-hand protein			**↓**			**↑**			**×**		*L. major*		*L. major*		Dillon et al., 2015; Amiri-Dashatan et al., 2020b	

Common genes found in independent transcriptomic and proteomic data obtained during Leishmania metacyclogenesis (procyclic vs. metacyclic promastigotes). We compared every differentially expressed gene found in different independent transcriptomic and proteomic analyses to find correlations in trends of mRNA and protein levels during metacyclogenesis. Results based on the search of 111 differentially expressed proteins (DEPs) (65 up regulated and 46 down regulated upon metacyclogenesis) against 3704 differentially expressed genes (DEGs) (1804 upregulated and 1900 downregulated upon metacyclogenesis). P → M – procyclic to metacyclic promastigote differentiation; **↑** mRNA or protein levels increase in procyclic to metacyclic differentiation; ↓ - mRNA or protein levels decrease in procyclic to metacyclic differentiation; Correlation – whethe mRNA and protein levels both decrease or increase during procyclic to metacyclic differentiation. Gene IDs were all originally available based on the L. major genome Although some of these data are related to other species, it was the authors choice to present them in a particular way, and we kept them in their original presentation and decided to use the L. major genome as a reference for all analyses.

For the stress response, two peroxidases (LmjF.26.0800 and LmjF.15.1040) appeared to be upregulated at both the mRNA and protein levels in metacyclic promastigotes in comparison to procyclic promastigote forms. This may represent an adaptation of the parasite during metacyclogenesis, preparing to infect mammalian cells. A putative gene (LmjF.12.1130 - NADH/flavin oxidoreductase) and a heat shock protein (LmjF.33.2390 - TRAP1/HSP75) did not present correlation between protein and mRNA levels, with both showing a decrease in transcript levels in metacyclic promastigotes and an increase in protein levels (**Table 1**).

During metacyclogenesis, correlation between mRNA and protein levels was observed for genes encoding for mitochondrial proteins: two cytochrome c oxidase subunits (LmjF.26.1710 and LmjF.25.1130) appeared downregulated in metacyclic promastigotes compared to procyclic promastigotes (**Table 1**).

The most expressive finding for metacyclogenesis was for gene expression-related genes. Of the 11 related genes found in our analysis, 8 (over 72%) were found to consistently be downregulated (both mRNA and proteins) in metacyclic promastigotes. This category included elongation factors (LmjF.18.0740, LmjF.36.0180, LmjF.34.0840), RNA helicases (LmjF.21.1552, LmjF.35.3100), histone H3 (LmjF.10.0870), guanosine monophosphate (GMP) reductase (LmjF.17.0725) and the protein transport protein SEC13 (LmjF.32.0050). This downregulation in gene expression-related proteins is in accordance with the previously demonstrated reduction in transcription and translation in metacyclic promastigotes (Kloehn et al., 2015).

Proteins involved in cell energy metabolism, such as phosphoglycerate kinase (PGKC) (LmjF.20.0100) and pyruvate kinase (LmjF.35.0030), were increased in both mRNA and protein levels during metacyclogenesis (**Table 1**). On the other hand, ATP-dependent 6-phosphofructokinase (PFK) (LmjF.29.2510), a main regulator of glycolysis (Mor et al., 2011), showed decreased levels during metacyclogenesis in *L. tropica* (**Table 1**), possibly indicating a reduction in glycolytic activity. These data, with reduced levels of cytochrome c oxidase subunits V and VII, corroborate the hypothesis that cell respiration is affected upon metacyclogenesis. Additionally, greater energy consumption has been shown to occur in procyclic promastigotes of *L. mexicana* than in metacyclic promastigotes due to a higher replication rate (Costa et al., 2011).

Interestingly, glutamate dehydrogenase (GDH) (LmjF.28.2910) shows opposite trends in different *Leishmania* species In *L. major*, both mRNA and protein levels of GDH increased in metacyclics in comparison to procyclic promastigotes. In *L. tropica*, however, there was a decrease in GDH (LmjF.28.2910) during metacyclogenesis at both protein and mRNA levels. Nonetheless, another GDH (LmjF.15.1010) presented no correlation when we compared *L. major* and *L. tropica* data, with decreased mRNA levels but increased protein levels (**Table 1**).

A putative lipophosphoglycan biosynthetic protein 3 (LPG3) is reportedly downregulated in metacyclic forms of *L. tropica* and *L. major* (Inbar et al., 2017; Amiri-Dashatan et al., 2020a). LPG is a protein responsible for insect midgut binding and also acts as a virulence factor in mammalian hosts. This supports the idea that regulation of LPG expression is stage-specific, as is the ability of *Leishmania* to bind to the host midgut. (Pimenta et al., 1992; Sacks and Kamhawi, 2001; Wilson et al., 2010). Metacyclic promastigotes, however, present upregulation of transferase mRNAs, in accordance with the previous identification of elongated LPG in this specific phase (Rowton et al., 1995). Adding to this hypothesis, mRNA for the glycosyltransferase gene, which is involved in elongation and modification of LPG, appears to be upregulated in nectomonads (Coutinho-Abreu et al., 2020). Other transferases (galactosyl and mannosyl-transferases) are also upregulated in nectomonad and metacyclic promastigotes, indicating that these stages of metacyclogenesis detach from the midgut, in accordance with what is known about the parasite life cycle.

Overall, of 46 common genes found in our independent analysis of transcriptomic and proteomic data, more than 60.9% (28 genes) presented a positive correlation between mRNA and protein levels in metacyclogenesis; the other 39.1% (18 genes) exhibited contrary trends (**Table 1**). Based on these findings, the data obtained from each individual study supports the hypothesis that transcriptional differences exist between procyclic and metacyclic forms in the *Leishmania* life cycle, even though the genome is mostly constitutively expressed.

## Amastigogenesis

Amastigote differentiation or amastigogenesis is the process by which metacyclic promastigotes differentiate into amastigotes inside phagocytic cells. Amastigotes live and replicate inside mammalian host cells in the compartment called parasitophorous vacuoles (PVs) or phagolysosomes (**Figure 1**), which is the most drastic change in environmental conditions faced by the parasite in its life cycle, involving pH, temperature, and nutrient availability changes, as previously described (Gupta et al., 2001). This environmental alteration causes some observable consequences on parasite gene expression, even though the vast majority of genes appear to be constitutively expressed (Cohen-Freue et al., 2007). An overall reduction in RNA content is observed in amastigotes compared to promastigotes (Shapira et al., 1988). Indeed, most differences in mRNA abundance are observed when comparing the amastigote and procyclic promastigote stages (Inbar et al., 2017). A study showed that RNA abundance seems to play an important role in the early stages of amastigogenesis, but later in the process, posttranscriptional and translational mechanisms act to regulate gene expression (Lahav et al., 2011). The same study found that approximately 20-30% of genes presented a correlation between mRNA and protein levels during differentiation, most of which were up/downregulated in the early stages of differentiation (Lahav et al., 2011).

Amastigogenesis can be induced *in vitro* for *Leishmania* species by subjecting promastigotes to environmental conditions that mimic the inside of the mammalian host cell (37°C, pH ~ 5.0) for over 5 h (Barak et al., 2005; Zilberstein, 2020). In this context, temperature seems to play an important role in altering the gene expression and morphology of the parasite, inducing differentiation (Zilberstein and Shapira, 1994; Kramer et al., 2008; Alcolea et al., 2010b; Zilberstein, 2020). Although axenic amastigotes have been proven to be good models for amastigote studies, they lack complex host signaling and downstream effects (Saar et al., 1998; Gupta et al., 2001; Barak et al., 2005). RNA expression studies have revealed transcriptional differences between axenic amastigotes and intracellular amastigotes related to metabolic processes, surface proteins, intracellular transport and response to oxidative stress (Rochette et al., 2009).

A remarkable change in amastigote morphology is the flagellum structure. The paraflagellar rod structure is known to compose the flagellum in kinetoplastids during specific flagellated life stages; its absence has been observed in amastigotes (Portman and Gull, 2010). Accordingly, several studies have identified upregulation of paraflagellar rod protein 1 and 2 mRNAs (PRF1 and PRF2) in promastigotes of approximately 10- to 15-fold higher than in amastigotes (Moore et al., 1996; Mishra et al., 2003). PRF genes encode a component of the paraflagellar rod.

Another change involves the transport of sugars in promastigote and amastigote metabolism. It is known that the main carbon source for promastigotes is sugar and amino acids but that amastigotes mainly utilize amino acids and fatty acids (Krassner, 1969; Krassner and Flory, 1972; Hart and Coombs, 1982; Kuile and Opperdoes, 1992; Opperdoes and Coombs, 2007). A transcriptomic analysis confirmed that levels of different sugar and amino acid transporter genes are upregulated in procyclic promastigotes in comparison to amastigotes (Inbar et al., 2017). In metacyclogenesis, the mRNA levels of these transporters further increased from procyclic promastigotes to neptomonads, reaching their peak in the metacyclic stage (Inbar et al., 2017). This is strong evidence that observable metabolic changes during the *Leishmania* life cycle can be detected at the transcriptomic level.

Moreover, there are similarities between the transcriptional profile of metacyclic promastigotes and amastigotes (for example, in comparison to procyclics, metacyclic promastigotes present an upregulation of amastin-like proteins - known to be characteristic of the amastigote phase of *Leishmania*), suggesting that metacyclogenesis is a process that “prepares” the parasite for infection (Inbar et al., 2017).

To assess correlations between mRNA and protein levels in amastigogenesis, we analyzed data from 8 articles involving five *Leishmania* species: 5 contained transcriptomic data (Almeida et al., 2004; Holzer et al., 2006; Leifso et al., 2007; Saxena et al., 2007; Alcolea et al., 2010a) and 3 proteomic data (Walker et al., 2006; Leifso et al., 2007; Brotherton et al., 2010). These studies obtained *Leishmania* amastigotes in different ways: recovered from BALB/c mice lesions (Almeida et al., 2004; Holzer et al., 2006, and Leifso et al., 2007), macrophage lysis (Alcolea et al., 2010a) and *in vitro* cultivation of axenic amastigotes (Walker et al., 2006; Saxena et al., 2007 and Brotherton et al., 2010). The analysis was performed in a similar manner as previously described for metacyclogenesis.

In these works, differentially expressed mRNAs and proteins were compared and correlations were established (**Table 2**). Each gene listed in **Table 2** was identified as differentially expressed in the analyzed proteomic or transcriptomic studies comparing promastigote to amastigote differentiation. Following the same trend observed for promastigotes, a positive correlation between mRNA and protein levels was found for most genes in **Table 2** although some genes presented opposite levels. We analyzed genes within the following biological groups: stress response, gene expression, energy metabolism, cell signaling and proliferation. Hypothetical proteins and other biological functions were also considered (**Table 2**).

**Table 2:** Common genes found in independent transcriptomic and proteomic data obtained from amastigogenesis (promastigote vs. amastigote) differentiation.

Biological group	GeneID	Protein	mRNA levels(P→ A)	Protein levels(P→ A)	Correlation	Species (mRNA data)	Species (protein data)	References
Stress response	LmjF.28.2780	HSP70 heat-shock protein hsp70	**↑**	**↑**	✔	*L. major*	*L. infantum*	Almeida et al., 2004; Brotherton et al., 2010
	LmjF.28.2781	HSP70 heat-shock protein hsp71	**↓**	**↑**	**×**	*L. major*	*L. infantum*	Leifso et al., 2007; Brotherton et al., 2010
	LmjF.30.1540	Flavoprotein-like protein	**↑**	**↑**	✔	*L. mexicana*	*L. infantum*	Holzer et al., 2006; Brotherton et al., 2010
	LmjF.32.1820	Superoxide dismutase	**↓**	**↑**	**×**	*L. major*	*L. infantum*	Leifso et al., 2007; Brotherton et al., 2010
	LmjF.32.1940	Chaperone protein DNAJ/DnaJ homolog – JDP7	**↑**	**↑**	✔	*L. major*	*L. infantum*	Almeida et al., 2004; Brotherton et al., 2010
	LmjF.33.0312	HSP83 heat shock protein 83−1	**↓**	**↓**	✔	*L. major, L.donovani*	*L. infantum*	Leifso et al., 2007; Saxena et al., 2007; Brotherton et al., 2010
	LmjF.36.0070	stress-inducible protein STI1 homologue	**↓**	**↓**	✔	*L. mexicana, L. donovani*	*L. infantum*	Holzer et al., 2006; Saxena et al., 2007; Brotherton et al., 2010
	LmjF.36.2030	Chaperonin HSP60, mitochondrial	**↓**	**↑**	**×**	*L. donovani*	*L. infantum*	Leifso et al., 2007; Saxena et al., 2007
Gene Expression	LmjF.30.3520	Adenosylmethionine synthase	**↓**	**↑**	**×**	*L. major*	*L. infantum*	Almeida et al., 2004; Brotherton et al., 2010
	LmjF.35.5040	poly(a)-binding protein	**↓**	**↓**	✔	*L. major*	*L. infantum*	Leifso et al., 2007; Brotherton et al., 2010
	LmjF.35.5041	poly(a)-binding protein	**↑**	**↓**	**×**	*L. donovani*	*L. infantum*	Saxena et al., 2007; Brotherton et al., 2010
	LmjF.25.0490	RNA-binding protein UBP1	**↓**	**↓**	✔	*L. major*	*L. infantum*	Leifso et al., 2007; Brotherton et al., 2010
	LmjF.19.0030	Histone H2B	**↓**	**↓**	✔	*L. major*	*L. infantum*	Leifso et al., 2007
	LmjF.03.0980	Elongation initiation factor 2 alpha subunit	**↓**	**↑**	**×**	*L. infantum*	*L. infantum*	Alcolea et al., 2010a; Brotherton et al., 2010
	LmjF.10.0970	Histone h3	**↓**	**↑**	**×**	*L. mexicana*	*L. infantum*	Holzer et al., 2006; Leifso et al., 2007
	LmjF.35.0370	ATP-dependent DEAD-box RNA helicase	**↓**	**↑**	**×**	*L. major*	*L. infantum*	Almeida et al., 2004; Brotherton et al., 2010
	LmjF.02.0020	Histone H4	**↓**	**↓**	✔	*L. mexicana*	*L. infantum*	Holzer et al., 2006; Leifso et al., 2007
	LmjF.06.0010	Histone H4	**↓**	**↓**	✔	*L. mexicana*	*L. infantum*	Holzer et al., 2006; Leifso et al., 2007
	LmjF.31.3180	Histone H4	**↓**	**↓**	✔	*L. mexicana*	*L. infantum*	Holzer et al., 2006; Leifso et al., 2007
	LmjF.35.1310	Histone H4	**↓**	**↓**	✔	*L. mexicana*	*L. infantum*	Holzer et al., 2006; Leifso et al., 2007
	LmjF.36.0020	Histone H4	**↓**	**↓**	✔	*L. mexicana*	*L. infantum*	Holzer et al., 2006; Leifso et al., 2007
	LmjF.25.2450	Histone H4	**↓**	**↓**	✔	*L. major*	*L. infantum*	Almeida et al., 2004; Leifso et al., 2007
	LmjF.35.3860	t-Complex protein 1, eta subunit, putative	**↓**	**↓**	✔	*L. donovani, L. major*	*L. infantum*	Leifso et al., 2007; Saxena et al., 2007; Brotherton et al., 2010
Energy metabolism	LmjF.23.0690	3-ketoacyl-coa thiolase-like protein	**↓**	**↓**	✔	*L. infantum*	*L. infantum*	Alcolea et al., 2010a; Brotherton et al., 2010
	LmjF.21.1770	ATP synthase F1 subunit gamma protein	**↓**	**↓**	✔	*L. mexicana*	*L. infantum*	Holzer et al., 2006; Brotherton et al., 2010
	LmjF.30.2970	glyceraldehyde 3-phosphate dehydrogenase, glycosomal	**↓**	**↓**	✔	*L. major*	*L. infantum*	Leifso et al., 2007; Brotherton et al., 2010
	LmjF.36.1260	Fructose 1,6-bisphosphate aldolase	**↓**	**↑**	**×**	*L. mexicana*	*L. panamensis*	Holzer et al., 2006; Walker et al., 2006
	LmjF.14.1160	Enolase	**↓**	**↓**	✔	*L. mexicana, L. donovani*	*L. infantum*	Holzer et al., 2006; Leifso et al., 2007; Saxena et al., 2007
	LmjF.31.1630	putative 3-ketoacyl-coa thiolase-like protein	**↓**	**↑**	**×**	*L. infantum*	*L. infantum*	Alcolea et al., 2010a; Brotherton et al., 2010
Cell signaling	LmjF.31.1630	PGFS prostaglandin f2-alpha synthase	**↓**	**↑**	**×**	*L. infantum*	*L. infantum*	Alcolea et al., 2010a; Brotherton et al., 2010
	LmjF.36.0550	CRK1, cell division protein kinase 2	**↓**	**↓**	✔	*L. mexicana, L. donovani*	*L. infantum*	Holzer et al., 2006; Saxena et al., 2007; Brotherton et al., 2010
	LmjF.25.0910	Cyclophilin a	**↓**	**↓**	✔	*L. major*	*L. infantum*	Leifso et al., 2007
	LmjF.29.0880	ADP-ribosylation factor-like protein	**↓**	**↓**	✔	*L. mexicana*	*L. infantum*	Holzer et al., 2006; Leifso et al., 2007
Hypothetical proteins	LmjF.08.0860	Hypothetical protein, unknown function	**↑**	**↑**	✔	*L. donovani*	*L. infantum*	Saxena et al., 2007; Brotherton et al., 2010
	LmjF.34.0010	Short chain dehydrogenase	**↓**	**↑**	**×**	*L. mexicana*	*L. infantum*	Holzer et al., 2006; Brotherton et al., 2010
	LmjF.33.0610	paraflagellar rod component PFC16	**↓**	**↓**	✔	*L. major*	*L. infantum*	Leifso et al., 2007
Other	LmjF.17.0870	Meta 2 protein, putative	**↓**	**↓**	✔	*L.mexicana, L. major, L. donovani*	*L. infantum*	Holzer et al., 2006; Leifso et al., 2007; Saxena et al., 2007
	LmjF.33.0820	Beta-tubulin	**↓**	**↑**	**×**	*L. major*	*L. panamensis*	Walker et al., 2006; Leifso et al., 2007
	LmjF.10.0460	GP63, leishmanolysin	**↓**	**↓**	✔	*L. major*	*L. infantum*	Leifso et al., 2007
	LmjF.10.0470	GP63, leishmanolysin	**↓**	**↓**	✔	*L. major*	*L. infantum*	Leifso et al., 2007
	LmjF.16.1430	Paraflagellar rod protein 2C	**↓**	**↓**	✔	*L. mexicana, L. major*	*L. infantum*	Holzer et al., 2006; Leifso et al., 2007
	LmjF.29.1750	Paraflagellar rod protein 2C	**↓**	**↓**	✔	*L. mexicana*	*L. infantum*	Holzer et al., 2006; Leifso et al., 2007
	LmjF.29.1760	Paraflagellar rod protein 2C	**↓**	**↓**	✔	*L.donovani, L. major*	*L. infantum*	Leifso et al., 2007; Saxena et al., 2007

Common genes in independent transcriptomic and proteomic data obtained during Leishmania promastigote to amastigote differentiation. We compared every differentially expressed gene in different independent transcriptomic and proteomic analyses to find correlations in trends of mRNA and protein levels during amastigogenesis. P → A – promastigote to amastigote differentiation; ↑ - mRNA or protein levels increase in promastigote to amastigote differentiation; ↓ - mRNA or protein levels decrease in promastigote to amastigote differentiation; Correlation – whether mRNA and protein levels are both decreasing or increasing during promastigote to amastigote differentiation. For gene IDs originally provided in other species’ codes, the L. major Friedlin syntenic ortholog was obtained at TriTrypDB for the comparison analysis. In the few cases where there was no synteny, we utilized nonsyntenic orthologs that encoded the same protein for comparison.

Stress response proteins appeared upregulated in amastigotes compared to promastigotes in response to the drastic environmental changes that trigger differentiation and the hostile acidic PV environment. mRNA and protein levels of stress response proteins, such as HSP70 (LmjF.28.2780) and Chaperone protein DNAJ homolog – JDP7 (LmjF.32.1940), presented a positive correlation and were consistently upregulated in amastigotes. On the other hand, superoxide dismutase (LmjF.32.1820) showed opposite mRNA and protein levels, with a decrease in mRNA but an increase in protein. It is interesting to note that some HSPs (LmjF.28.2780 and LmjF.33.0312) presented correlations but that others (LmjF.28.2781 and LmjF.36.2030) did not. Stress inducible protein 1 (STI1) transcripts are heat inducible in *L. major* promastigotes, with increased transcript levels when subjected to an increased temperature (Webb et al., 1997). Our analysis revealed a decrease in both the mRNA and protein content of an STI1 homolog in amastigotes of *L. mexicana* and *L. donovani* (mRNA data) and *L. infantum* (protein data).

Gene expression-related genes appeared mostly downregulated, with mRNA and protein levels of 10 out of 14 genes being consistently downregulated in amastigotes, suggesting that gene expression is reduced in this life stage. In this category, we observed a polyA-binding protein (LmjF.35.5040), an RNA upstream-binding protein (UBP2) (LmjF.25.0490), and several histones. Histone H4 was the most notable observation, with 6 different genes being downregulated at both the mRNA and protein levels in amastigotes (LmjF.02.0020, LmjF.06.0010, LmjF.31.3180, LmjF.35.1310, LmjF.36.0020 and LmjF.25.2450). Reduced mRNA and protein levels histone 2B (LmjF.19.0030) also was detected in amastigotes, but histone 3 (LmjF.10.0970) mRNA and protein levels did not correlate.

For energy metabolism-related proteins, fructose 1,6-bisphosphate aldolase (LmjF.36.1260) and putative 3-ketoacyl-CoA thiolase-like protein (LmjF.31.1630) were the only proteins, of 6 in total, that were upregulated in amastigotes. These are coincidently the only two that did not present mRNA and protein level correlations, as their mRNAs were downregulated. For all 4 other proteins (LmjF.23.0690, LmjF.21.1770, LmjF.30.2970, LmjF.14.1160), both mRNA and protein levels were reduced upon amastigogenesis. The consistent downregulation of glycolytic enzymes (LmjF.30.2970 - glyceraldehyde 3-phosphate dehydrogenase and LmjF.14.1160 – Enolase) is in accordance with what has been described for *L. mexicana* amastigotes, utilizing less glucose in relation to promastigotes (Hart and Coombs, 1982).

It is also possible to observe some classical developmentally regulated genes, such as several GP63 and a few paraflagellar rod protein genes (PFR2). Both the protein and mRNA levels of these genes decrease during amastigote differentiation, in accordance with what is known about the parasite life cycle, as discussed previously.

One interesting trend unveiled by **Table 2** is that of the 30 genes that presented mRNA and protein correlations, 86% (26 genes) showed decreasing expression in amastigotes. This is consistent with what has been reported about a decrease in mRNA and protein content in amastigotes (Shapira et al., 1988). Of the only 4 correlating genes in the table with increased expression during amastigogenesis, 3 are related to the stress response, and the other is a hypothetical protein.

Overall, of 43 common genes found in our analysis of independent transcriptomic and proteomic data, 30 (69.8%) presented positive correlations between mRNA and protein levels during amastigote differentiation; opposite trends in mRNA and protein levels were found for the other 13 genes (30.2%). Once again, this supports the idea that mRNA and protein levels can correlate in the *Leishmania* life cycle. This finding of over 69% correlation between mRNA and protein trends in amastigote differentiation is larger than the 53% previously observed in combined proteomic and transcriptomic studies of *L. infantum* differentiation (McNicoll et al., 2006). Our analysis, however, incorporated a larger number of *Leishmania* species and several independent studies, which might explain the difference in the percentage of mRNA/protein correlations.

## Conclusion

**Figure 4:**
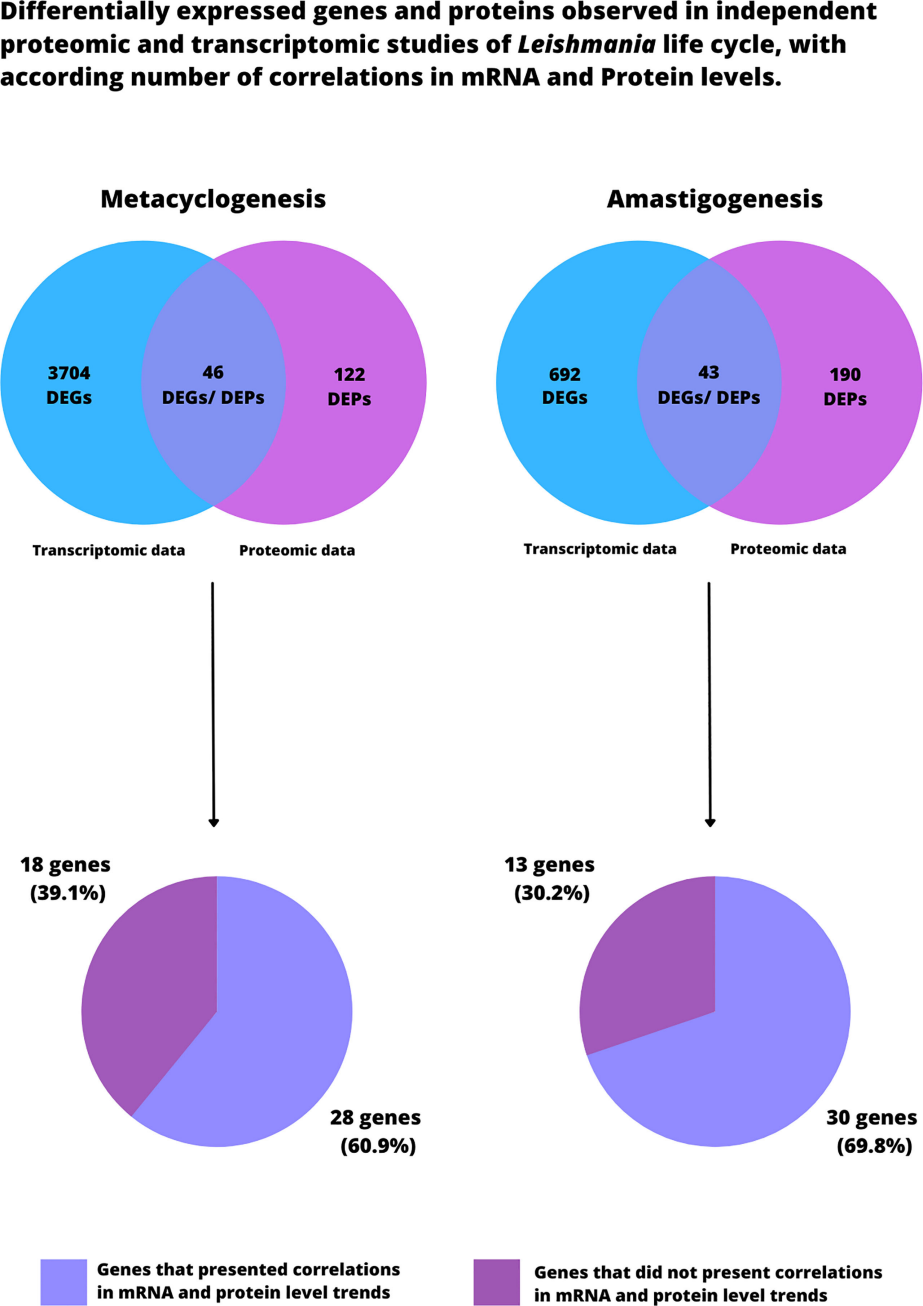
Differentially expressed genes and proteins observed in independent transcriptomic and proteomic studies of *Leishmania* life cycle. Both Venn Diagrams on the top portion of the figure describe the total number of DEPs and DEGs found in all the analyzed studies that was based this review. Further, among the commonly differentially expressed genes, we indicate the amount of genes that presented correlation between mRNA and protein levels in metacyclogenesis and amastigogenesis. It is worth noting that these results should be read as proof-of-concept, since no statistical analysis was performed to compare the analyzed studies.

It is important to point that this review was based on an extensive literature review and manual *ad-hoc* curation. No statistical analysis was performed with the data from all the analyzed studies, proposing this reading as a proof-of-concept. Our analyses were obtained from over 4,000 genes in transcriptomic data and over 400 proteins in proteomic data during metacyclogenesis and amastigonesis in *Leishmania*. Interestingly, only 46 and 43 genes were commonly differentially expressed in metacyclogenesis and amastigogenesis, respectively (**Figure 4**). Among these, 28 and 30 genes presented correlation between mRNA and protein levels in metacyclogenesis and amastigonesis, respectively (**Figure 4**). The considered genes that did not present correlation corroborated the idea that it is not always possible to accurately predict protein levels based solely on levels of mRNA, mostly due to extensive posttranscriptional mechanisms regulating gene expression. In particular case of our analysis the lack of correlation might be related to the different *Leishmania* species being compared that may interfere in establishing such correlations.

The idea that the *Leishmania* genome is constitutively expressed at the transcriptional level does not necessarily mean that protein levels cannot follow the same trend of the corresponding mRNA to be expressed. Here, we showed that the mRNA and protein levels of several genes increase or decrease concomitantly during metacyclogenesis and amastigogenesis. These differences in mRNA and protein levels might also be used in epidemiological practice and/or research as stage-specific markers to identify, isolate and recognize specific life forms in the *Leishmania* life cycle.

To date, little research establishing the correlation of mRNA and protein levels in *Leishmania* has been performed due to the complexity of data handling. The increase in proteomic and transcriptomic data associated with detailed comparative analysis will certainly enrich the understanding of gene expression regulation in trypanosomatids, providing new ways to use molecular biological data in the control and treatment of the disease. Another approaches that may be helpful in obtaining new answers for the *Leishmania* gene expression questions could be performed through half-lives measurement (Archer et al., 2008) and polysome profiling (Bifeld et al., 2018; Karamysheva et al., 2018). This technique may provide important information on the correlation between mRNA and protein levels in *Leishmania*, by exclusively analyzing mRNAs that are in fact being translated.

## Author Contributions

LC, JA, and LF-W contributed to the conception of the review and manuscript revision. LC made the first draft, performed analysis, and designed figures and tables. JA and LF-W added new information to the original manuscript. All authors contributed to the article and approved the submitted version.

## Funding

The work was supported by São Paulo Research Foundation (FAPESP) - grants #2017/23696-1 (LC), #2021/04422-3 (JA) and #2018/23512-0 (LF-W).

## Conflict of Interest

The authors declare that the research was conducted in the absence of any commercial or financial relationships that could be construed as a potential conflict of interest.

The handling editor LT declared a shared affiliation with the authors at the time of review.

## Publisher’s Note

All claims expressed in this article are solely those of the authors and do not necessarily represent those of their affiliated organizations, or those of the publisher, the editors and the reviewers. Any product that may be evaluated in this article, or claim that may be made by its manufacturer, is not guaranteed or endorsed by the publisher.
